# Microscopic polyangiitis initially presenting with idiopathic pulmonary fibrosis: a case report

**DOI:** 10.3389/fmed.2023.1157922

**Published:** 2023-05-24

**Authors:** Chi Shao, Ruxuan Chen, Hui Huang, Yang Zhao, Keqi Chen, Kai Xu

**Affiliations:** ^1^Department of Pulmonary and Critical Care Medicine, Peking Union Medical College Hospital, Chinese Academy of Medical Sciences and Peking Union Medical College, Beijing, China; ^2^Department of Radiological, Peking Union Medical College Hospital, Chinese Academy of Medical Sciences and Peking Union Medical College, Beijing, China

**Keywords:** usual interstitial pneumonia pattern, ANCA antibody, microscopic polyangiitis, antifibrotic therapy, idiopathic pulmonary fibrosis

## Abstract

Usual interstitial pneumonia is the most common type of microscopic polyangiitis (MPA)-associated interstitial lung disease, and patients may initially present with isolated pulmonary fibrosis, which often leads to a misdiagnosis of idiopathic pulmonary fibrosis (IPF). Here, we describe a patient who developed fever of unknown origin, microscopic hematuria and renal insufficiency, who then tested positive for antineutrophil cytoplasmic antibody (ANCA) and was diagnosed with MPA after receiving antifibrotic medication for IPF (original diagnosis) for almost 10 years. The patient's symptoms were ameliorated after administration of additional glucocorticoids and immunosuppressants.

## Introduction

Pulmonary fibrosis and diffuse alveolar hemorrhage were the main respiratory involvement of microscopic polyangiitis (MPA) (1), and usual interstitial pneumonia (UIP) pattern is the most common radiological manifestation (2). Interstitial lung disease (ILD) might be the first clinical manifestation in one fifth antineutrophil cytoplasmic antibodies (ANCA)-associated vasculitis (AAV), however, ILD might be diagnosed earlier than AAV in more than 80% p-ANCA positive patients (2). Patients with MPA may initially present with isolated pulmonary fibrosis, which often leads to a misdiagnosis of idiopathic pulmonary fibrosis (IPF).

## Case presentation

A 69-year-old male patient visited our hospital on December 1, 2021, due to dry cough and shortness of breath after activity for the past 10 years and intermittent fever for the past 3 months. In early November 2011, the patient suffered from a processive cough after catching a cold and was given antibiotics and cough suppressants, but no marked effect was observed. He also had shortness of breath after intense activities, which affected his daily work but did not affect his daily life. Therefore, he did not seek further medical consultation. In early January 2012, the above symptoms were aggravated by a cold, accompanied by a small amount of white sputum, without fever and hemoptysis. The cough was relieved after treatment with antibiotics and cough suppressants at a local hospital. However, shortness of breath was aggravated during intense activities. He presented to the local hospital again, and chest computed tomography (CT) showed pulmonary fibrosis.

**Figure 1:**
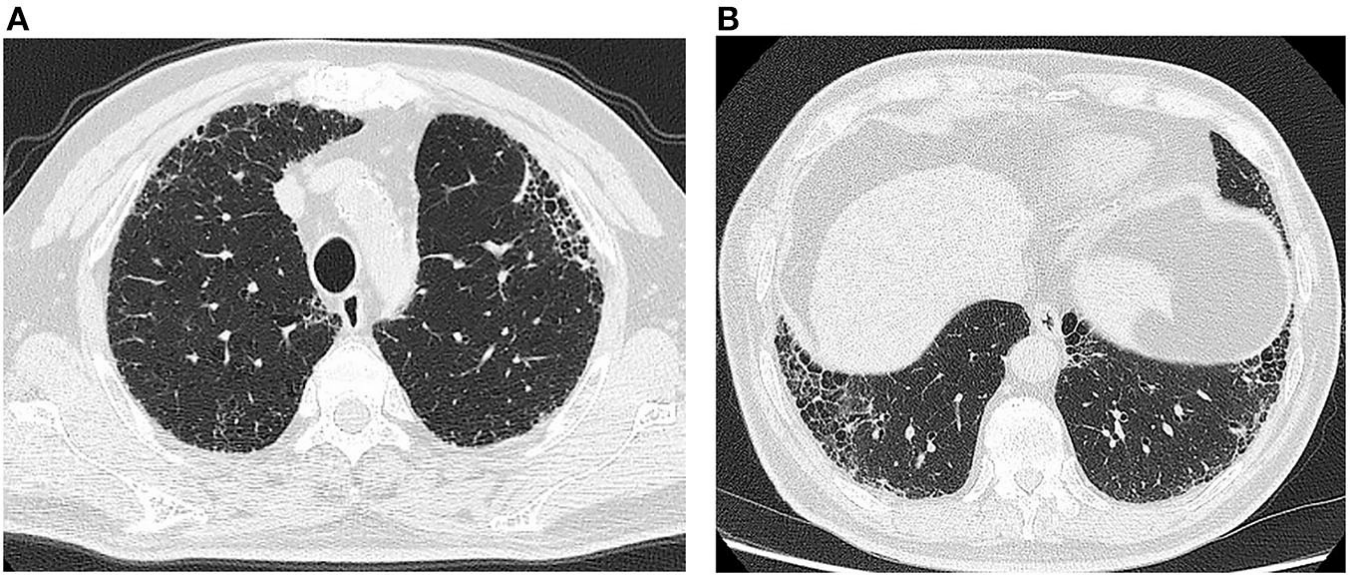
Chest HRCT: (May 8, 2012, **A, B**): Reticular and scattered honeycomb opacities predominantly in the proximal pleural parts of both lungs without significant exudative opacities.

On May 8, 2012, the patient presented to our interstitial lung disease clinic. The 17-item antinuclear antibody (ANA) profile, antineutrophil cytoplasmic antibody (ANCA) profile and rheumatoid arthritis (RA)-related antibody profile [including rheumatoid factor, antikeratin antibody (AKA), antiperinuclear factor antibody (APF) and anticyclic citrullinated peptide (CCP)] were all negative. The results of complete blood count testing, urine sediment and routine urine testing and biochemical analysis were all normal. The chest high-resolution CT (HRCT) manifestations were consistent with usual interstitial pneumonia (UIP) (Figure 1). The pulmonary function tests showed mild restrictive ventilation dysfunction and moderate diffusion impairment: forced expiratory volume in one second (FEV1)/forced vital capacity (FVC), 78%; FVC/FVC% predicted, 2.81 L/74.9%; total lung capacity (TLC)/TLC% predicted, 5.09 L/86.3%; diffusing capacity for carbon monoxide (DLCO)/DLCO% predicted, 3.66 mmol/min/kpa/52.9%. Bronchoalveolar lavage fluid tests showed the following: cell count, 8.6 × 10^6^/L; phagocytes, 64%; neutrophils, 11%; lymphocytes, 24%; eosinophils, 1%. After a global multidisciplinary discussion (MDD), he was diagnosed with idiopathic pulmonary fibrosis (IPF) and enrolled in the INPULSIS clinical trial (3). The patient took nintedanib (after unblinding, the patient was in the nintedanib group) from May 21, 2012, to June 15, 2017, i.e., in the INPULSIS and INPULSIS ON trials. During this period, he was complained with intermittent diarrhea, which was relieved with smectite and berberine. The series reports of PFTs were listed in Table 1.

**Table 1:** Series reports of pulmonary function tests.

**Characters**	**FEV1/FVC (%)**	**FVC (L)**	**FVC% predicted**	**TLC (L)**	**TLC% predicted**	**DLCO (mmol/min/kpa)**	**DLCO% predicted**
May 2012	78	2.81	74.9	5.09	86.3	3.66	52.9
May 2013	78.9	2.98	79.4	5.28	89.8	2.92	42.6
June 2017	75.6	2.71	73.1	4.98	79.8	3.02	42.8
December 2021	76.9	2.31	69	4.47	72	2.89	36

FEV1, forced expiratory volume in one second; FVC, forced vital capacity; TLC, total lung capacity; DLCO, diffusing capacity for carbon monoxide.

**Figure 2:**
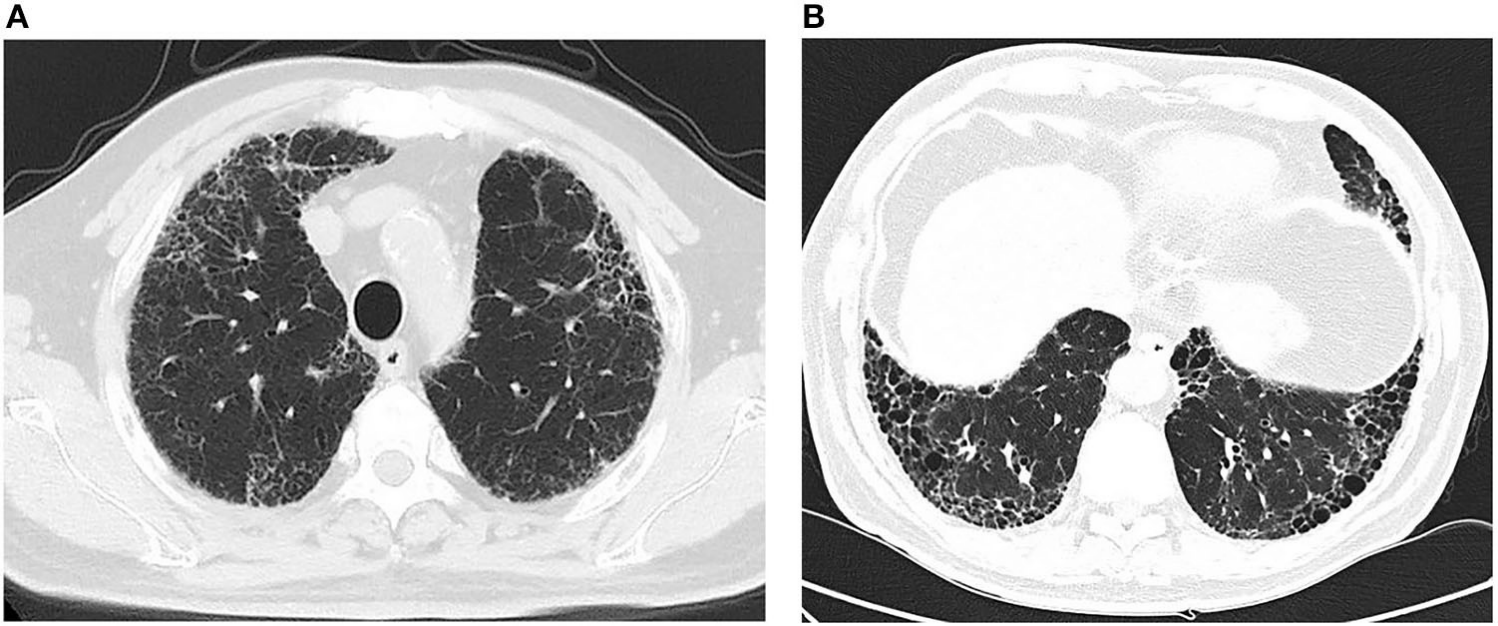
Chest HRCT: (September 27, 2021, **A, B**): Progressive Reticular and honeycomb opacities in the proximal pleural parts of both lungs compared with previous observations.

When the INPULSIS ON trial concluded, nintedanib was no longer free of charge. He therefore started pirfenidone (0.6 g tid) on January 13, 2018. No significant adverse effects were reported. However, since September 2019, he experienced worse exertional shortness of breath than before and was unable to perform physical labor. He complained of fatigue in early September 2021 and had a fever on September 12, with a maximum temperature that ranged from 37.8 to 38.7°C. Dry cough was aggravated, and tests for COVID-19 were negative. Repeated chest CT revealed pulmonary fibrosis progression but no significant exudative shadows (Figure 2). The microbiological analysis for induced sputum, 1,3-β-D-glucan test, Widal test, Weil-Felix test, mycoplasma pneumoniae antibodies, chlamydia antibody and Legionella antibodies were all negative. Empirical antibiotics (i.e., meropenem, ceftriaxone, moxifloxacin and azithromycin) were ineffective. Then, he came to our outpatient department on December 1, 2021. He had no arthralgia, no dry mouth and dry eyes. He was healthy before 2012 but had a history of smoking (10 cigarettes/day for 20 years) and had quit smoking in 2008. He was a farmer and had no history of occupational dust exposure. Physical examinations revealed the following: pulse oxygen saturation of 95% while breathing air, no palpable superficial cervical lymph nodes and no clubbing of the fingers, Velcro-like crackles in both lower lungs and no swelling in either inferior limb. Routine blood test: white blood cell count, 10.4 × 10^9^/L (*N* < 9.5); neutrophil percentage, 84.3% (*N* < 75%); hemoglobin, 107 g/L (N: 120–160). Urine sediment and urine routine testing: red blood cell count, 80/μL; 100% abnormal morphology. The 24 h urine protein was 0.3 g. Biochemical tests: serum creatine, 147 μmol/L; blood urea nitrogen (BUN), 9.34 mmol/L; erythrocyte sedimentation rate (ESR), 82 mm/h (*N* < 15); C-reactive protein (CRP), 25.6 mg/L (*N* < 8). The serum complement analysis and quantitative analysis of IgG, IgM and IgA were normal. The ANA profile: ANA 1:320 (AC-4), other indicators were negative; ANCA (P-type) 1:20, and myeloperoxidase (MPO): 148 RU/mL (*N* < 20). The rheumatoid arthritis (RA) antibody profile showed that the rheumatoid factor (RF) level was 169 IU/mL (*N* < 20). The anti-extractable nuclear antigen (ENA), CCP, AKA and APF were all negative. Repeated pulmonary function tests indicated an FVC/FVC% predicted of 2.31 L/69%, a TLC/TLC% predicted of 4.47 L/72% and a DLCO/DLCO% predicted of 2.89 mmol/min/kpa/36%, which were worse than the findings from 2012 at our hospital.

Concomitant with malignancies were common for IPF patients. So, common cancer screening tests had been arranged annually for him during the follow-up, series cancer antigen (CA) serum biomarkers, fecal occult blood, annual abdominal and urinary ultrasonography, and annual chest CT scan. There were no significant abnormal of these test and examinations before December 2021.

**Figure 3:**
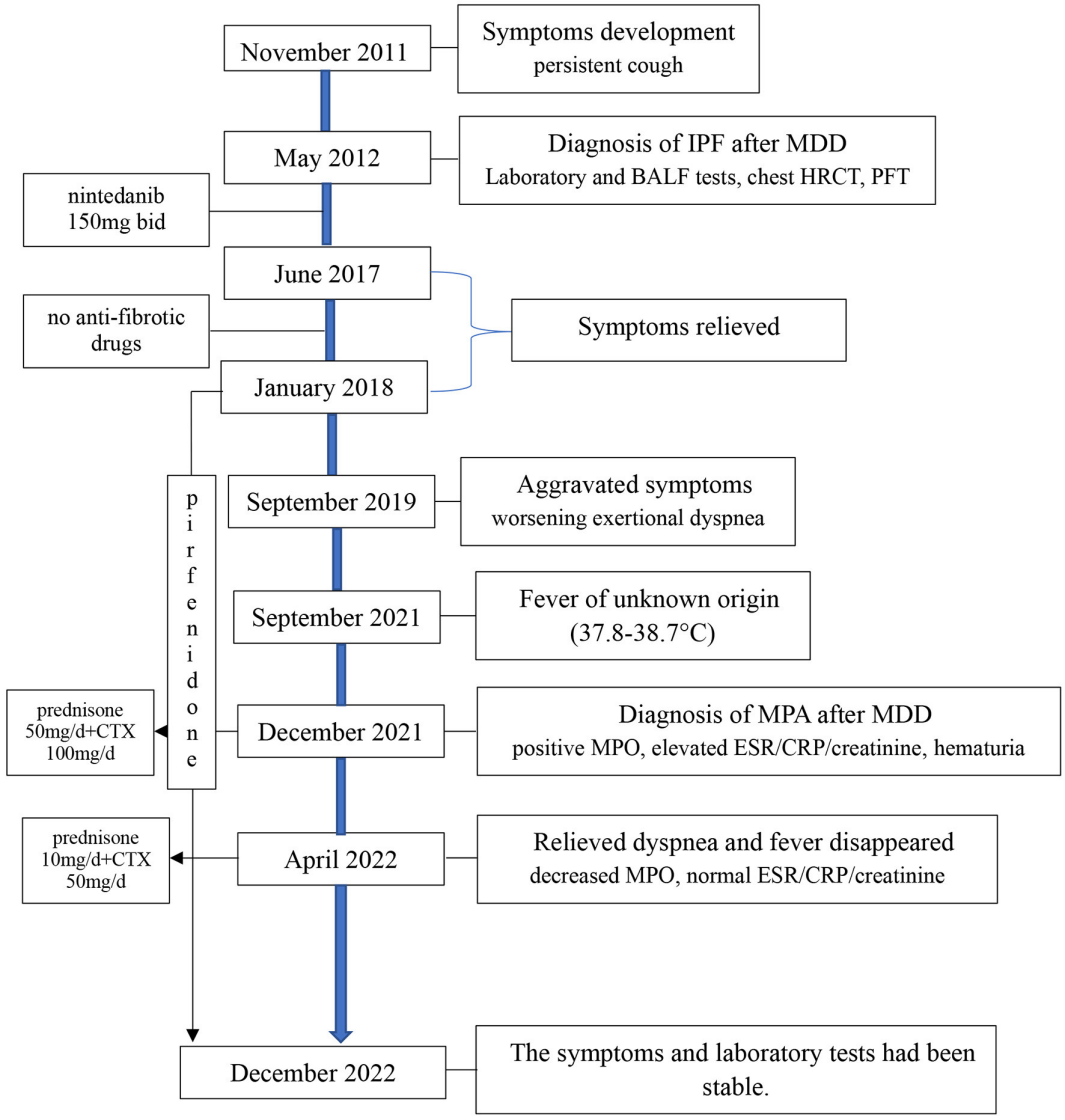
Chest HRCT: The timeline of our case. MDD, multidisciplinary discussion; BALF, bronchoalveolar lavage fluid; HRCT, high resolution CT; PFT, pulmonary function test; MPO, myeloperoxidase; ESR, erythrocyte sedimentation rate; CRP, C-reactive protein; CTX, cyclophosphamide.

According to his medical history, laboratory analysis and chest HRCT, he was diagnosed with microscopic polyangiitis (MPA) after repeated MDD, which included experts in ILD, rheumatology, nephrology and radiology. When he breathed deeply and held his breath, he suffered from severe dry cough. Renal biopsy was recommended but refused by the patient and his family. Then, the patient was given oral prednisone on December 20, 2021 (50 mg qd; the dose was reduced 3 weeks later) plus cyclophosphamide (100 mg qd) on the basis of pirfenidone. His temperature was normalized since Day 3 after administration, his fatigue was significantly relieved 2 weeks later, and his shortness of breath after activity was also alleviated. One month later (on February 2, 2022), repeated tests in local hospital showed an ESR of 28 mm/h, and his renal function and CRP had returned to normal. On April 19, 2022, he came to our clinic. His exertional shortness of breath relieved and repeated MPO was 34.5 RU/mL. The regimen of prednisone (10 mg qd), cyclophosphamide (50 mg qd) and pirfenidone (0.6 g tid) was maintained. The timeline was shown in Figure 3.

## Discussion

MPA, one type of systemic small-vessel vasculitis, frequently occurs in men aged 60–65 years and has been increasingly reported in the last two decades with the widespread use of the serum ANCA profile in clinical practice (4). In recent years, MPO-ANCA is considered to be possibly related to the occurrence and development of MPA, which has characteristic pathological manifestations of necrotizing small-vessel vasculitis and no or rare immune complex deposition in the vessel wall at the site of involvement. However, the specific etiology and pathogenesis of MPA remain unclear. Additionally, 95% of MPA patients have been reported to be positive for ANCA, approximately 70% of whom are positive for MPO-ANCA (5, 6). In MPA patients, the kidney is the most commonly involved organ, followed by the lungs. Pulmonary involvement in MPA mainly manifests as interstitial lung disease (ILD) and diffuse alveolar hemorrhage (7), and UIP-ILD is the most common chest HRCT phenotype of MPA-ILD. Patients with MPA-UIP often initially present with IPF and are diagnosed with MPA due to other manifestations associated with ANCA and MPA during subsequent visits. As reported by Fernandez et al. (7) patients with MPA-UIP can present with UIP 6–108 months earlier than other manifestations of MPA (7). Some patients with UIP-ILD are positive only for MPO-ANCA without other manifestations of organ involvement or systemic disease, and some authors believe that MPA cannot yet be diagnosed at this stage, but a close follow-up of urine sediment and routine urine testing is needed to identify early renal involvement and promptly prevent progression to acute/rapidly progressive renal insufficiency. Thompson et al. (8) concluded that ~25% of MPA-UIP cases may progress to MPA and therefore suggested that patients with MPA-UIP who do not meet the diagnostic criteria for MPA should be monitored monthly for urinary occult blood to improve their prognosis through early detection of renal involvement and update their diagnosis and treatment (8).

Here, we reported a rare case in which the patient developed fever of unknown origin, microscopic glomerular hematuria and elevated inflammatory indicators, including elevated ESR and CRP, followed by renal insufficiency and high-titer MPO-ANCA 2–3 months later. According to the 2022 Rheumatology classification criteria for microscopic polyangiitis (9), he could be diagnosed with MPA. However, he had been diagnosed with IPF and administrated with antifibrotic medications for almost 10 years. In the IPF cohort of Ando et al. (10) although only 4.9% of patients were positive for MPO-ANCA at the time of initial diagnosis, the positive rate of MPO-ANCA reached 14.8% during subsequent visits (10). So, in addition to ANCA screening at the time of initial diagnosis of IPF patients, the possibility of MPA rather than infectious diseases and acute exacerbation of IPF should be considered in the presence of fever and significant ESR and CRP elevations during follow-up. The serum ANCA profile, urine test and renal function should be screened promptly for early diagnosis.

No uniform recommendations are available for the treatment of MPA-UIP. Thompson et al. (8) at the Mayo Clinic proposed that glucocorticoids and immunosuppressants are not recommended for isolated MPO-ANCA-positive UIP without extrapulmonary involvements, but antifibrotic meidications can be used as appropriate. However, active glucocorticoids and immunosuppressants are suggested for MPA-UIP (8). French scholars Maillet et al. (11) also considered that immunosuppressive agents cannot improve the prognosis of MPA-UIP, but antifibrotic drugs may benefit these patients (11). In this case report, the patient was administered antifibrotic drugs until MPA was diagnosed, and no acute exacerbation of IPF was reported for up to 10 years despite progression indicated by chest CT and pulmonary function parameters, also verifying to some extent that nintedanib and pirfenidone can delay progression and improve the prognosis of MPA-UIP. After the patient developed fever, elevated inflammatory indicators and positivity for anti-MPO-ANCA, the updated diagnosis of MPA was made. His condition improved with glucocorticoids and immunosuppressive agents. Therefore, antifibrotic medications are also recommended for MPA-UIP without extrapulmonary involvement and systemic inflammation. Glucocorticoids plus immunosuppressants are suggested for these patients once MPA-related systemic manifestations emerge.

## Conclusions

Regular follow-up is important for all IPF patients. If they suffered from fever of unknown origin, microscopic hematuria, and/or abnormal ESR or CRP, MPA should be considered for the differential diagnosis. Except for antifibrotic drugs, add-on glucocorticoids and immunosuppressants were suggested for them with the updated diagnosis of MPA.

## Data availability statement

The raw data supporting the conclusions of this article will be made available by the authors, without undue reservation.

## Ethics statement

The studies involving human participants were reviewed and approved by the Institutional Review Board (IRB) of Peking Union Medical College Hospital (K2238). The patients/participants provided their written informed consent to participate in this study. Written informed consent was obtained from the individual(s) for the publication of any potentially identifiable images or data included in this article.

## Author contributions

HH is the guarantor of the content of the manuscript including the data and analysis. HH, CS, and RXC conceived and designed the study. CS, RXC, KX, and YZ performed the study. CS and KQC analyzed the data. HH and CS wrote the paper. All authors have read and agreed to the published version of the manuscript.
